# The Role of Natural Antioxidants in the Prevention of Dementia—Where Do We Stand and Future Perspectives

**DOI:** 10.3390/nu13020282

**Published:** 2021-01-20

**Authors:** Anamaria Jurcau

**Affiliations:** 1 Department of Psycho-Neurosciences and Rehabilitation, Faculty of Medicine and Pharmacy, University of Oradea, nr 1 Universitatii Street, 410087 Oradea, Romania; anamaria.jurcau@gmail.com; 2 Neurology Ward, Clinical Municipal Hospital “Dr. G. Curteanu”, nr 12 Corneliu Coposu Street, 410469 Oradea, Romania

**Keywords:** cognitive decline, Alzheimer’s disease, oxidative stress, natural antioxidants, polyphenols

## Abstract

Dementia, and especially Alzheimer’s disease (AD), puts significant burden on global healthcare expenditure through its increasing prevalence. Research has convincingly demonstrated the implication of oxidative stress in the pathogenesis of dementia as well as of the conditions which increase the risk of developing dementia. However, drugs which target single pathways have so far failed in providing significant neuroprotection. Natural antioxidants, due to their effects in multiple pathways through which oxidative stress leads to neurodegeneration and triggers neuroinflammation, could prove valuable weapons in our fight against dementia. Although efficient in vitro and in animal models of AD, natural antioxidants in human trials have many drawbacks related to the limited bioavailability, unknown optimal dose, or proper timing of the treatment. Nonetheless, trials evaluating several of these natural compounds are ongoing, as are attempts to modify these compounds to achieve improved bioavailability.

## 1. Introduction

The healing properties of certain plants and even of everyday diet, were recognized and used since ancient times. “Let food be thy medicine and thy medicine be thy food” is a famous (and overexploited) quote attributed to Hippocrates, the father of modern medicine [1].

In the early 19th century industrialization and better living conditions led to an increase of the lifespan, which reached from 43/45 years in 1840 to 79/85 years in 2015 for males and females, respectively [2]. However, the gain in life expectancy can be further broken down into “healthy life expectancy” and “years lived with disability”. In high-income countries the last 10–11 years are spent with disability, versus 7–9 years in lower income countries [3]. Conditions which lead to disability in old ages include many neurodegenerative diseases, among which dementia, and especially Alzheimer’s disease (AD), rank very high. 

Oxidative stress, defined as an imbalance between the production and accumulation of oxygen reactive species (ROS) in cells and tissues and the ability of a biological system to detoxify these reactive products, is significantly involved in the pathogenesis of these neurodegenerative diseases. Unfortunately, in the natural selection process individuals capable of producing large amounts of oxidants as antimicrobial defenses were favored at the expense of suffering greater collateral damage in diseases occurring beyond the age of reproduction [4].

## 2. Method

To highlight the importance of the topic, I first ran a search on Medline using “dementia”, “epidemiology”, and “cost” as keywords. Next, in order to gather relevant advances in research in the field of oxidative stress in Alzheimer’s disease (AD) and in the conditions considered to be risk factors for AD, I performed a literature review in Medline (PubMed) using “oxidative stress” “Alzheimer’s disease”, “cardiovascular disease”, “cerebrovascular disease”, “diabetes mellitus”, and “lifestyle” as keywords. Reference lists of existing reviews were searched for additional relevant articles. I used no specified inclusion or exclusion criteria. For the second part of the review, a similar search was performed using “dietary antioxidants”, “Alzheimer’s disease”, “stroke”, “hypertension”, “diabetes”, “diet” as keywords with a review of the reference list for relevant articles. 

## 3. Epidemiology and Cost of Dementia

Alzheimer’s disease as well as other forms of dementia such as vascular or multi-infarct dementia, have a growing prevalence and have escalated as the 5th cause of death worldwide and the third cause of death in the European region when considering both sexes and all ages [5]. The number of individuals living with dementia have more than doubled between 1990 and 2016 [6,7] when they have been around an estimated 45 million, a figure expected to increase to 74.7 million by 2030 and 131.5 million by 2050 [7,8].

Due to the lack of effective disease-modifying therapies for Alzheimer’s disease, these numbers will pose a huge burden on individuals who have dementia, their caregivers, and on the health systems [6]. AD is third most expensive disease in the United States after cancer and coronary heart disease [9]. The total global costs associated with dementia in 2010 were estimated at 604 billion US dollars, which is approximately 1% of the world’s gross domestic product [10]. These will rise rapidly from 1 trillion US dollars in 2018 to a projected $2 trillion in 2030 [11] with about 70% of these costs coming from Western Europe and the United States. Direct costs of Alzheimer’s disease and dementia in the US were 183 billion dollars in 2010 and are expected to increase to 1.1 trillion by 2050, while the estimated value of informal care was approximated at 202 billion dollars in 2010 [12].

Despite these figures, according to the World Health Organization registry, there are worldwide only 170 drugs in development for AD as opposed to 433 for diabetes and 6833 for cancer [11]. This may be due to the longer duration of trials, greater expenses, less well defined target biology, limited available biomarkers, as well as to the high risk of failure [13].

To date the only effective means to date to overcome the aforementioned expenses is to try to prevent dementia by managing the conditions shown to increase the risk of developing AD or other forms of dementia, among which vascular disease, diabetes mellitus, obesity, and physical inactivity are prominent [14]. A common characteristic of the pathogenesis of these conditions and the pathogenesis of dementia is the strong involvement of oxidative stress. This is not very far from the original view of Denham Harman in that free radicals accounted for cellular damage, genetic mutations, cancer, and the degenerative processes linked to old age [15].

## 4. Oxidative Stress in the Pathogenesis of Alzheimer’s Disease

Although Alzheimer’s disease pathogenesis has been dominated for decades by the amyloid cascade hypothesis, accumulating evidence has linked mitochondrial dysfunction and increased generation of ROS to the development of AD [16,17]. The nervous system is particularly vulnerable to oxidative stress due to a number of factors, such as [18,19,20]:

- the dependence on auto-oxidizable neurotransmitters (dopamine)
- the richness of neuronal membranes in polyunsaturated fatty acids, very vulnerable to free radical attack
- high calcium traffic across neuronal membranes and interference with ion transport
- the release of iron ions by damaged cerebral parenchyma
- the presence of modest antioxidant defenses, with low brain levels of catalase, glutathione peroxidase, vitamin E

The main ROS involved in neurodegeneration are superoxide anion (O_2_^−^), hydrogen peroxide (H_2_O_2_), and hydroxyl radical (HO). In addition, reactive nitrogen species, such as nitric oxide, also contribute to the oxidative damage, by reacting with hydrogen peroxide to produce peroxynitrite (ONOO^−^) [21].

Because in the brain most of the energy necessary to drive cellular reactions is generated in the mitochondria from aerobic oxidation of glucose, the mitochondria play a pivotal role in the generation of oxidative species [22]. Indeed, mitochondrial dysfunction is well documented in AD [23,24,25] even before the development of clinical symptoms or amyloid pathology [16,24]. Byproducts of oxidative phosphorylation in mitochondrial complexes I and III are mainly superoxide and hydrogen peroxide [26]. Manganese superoxide dismutase (Mn-SOD) in the mitochondria and Cu/Zn SOD in the cytoplasm convert superoxide into hydrogen peroxide, while H_2_O_2_ is further converted by catalase and glutathione peroxidases into water [21]. An additional protective mechanism is activation of nuclear factor erythroid-2-related factor 2 (Nrf2). This factor is normally bound to the cytoplasmic Kelch-like ECH associated protein 1 (KEAP1), which facilitates the ubiquitination and subsequent proteolysis of Nrf2. However, in the presence of oxidants, KEAP1 releases Nrf2, which is translocated to the nucleus and activates transcription of antioxidant enzymes and proteins generically known as antioxidant response elements (AREs) [27].

In addition, mitochondria act as a calcium buffer, attempting to normalize intracellular calcium levels [28]. Calcium efflux from the mitochondria is achieved by exchange for sodium or through the permeability transition pore (PTP) [29]. However, in calcium overload of the mitochondrial matrix calcium binds to the F1 subunit of the ATP synthase leading to its dissociation from a dimer to a monomer state and to the formation of the permeability transition pore [30]. Prolonged PTP opening leads to membrane swelling and rupture as well as to cytochrome c release triggering apoptosis [31]. In later stages of AD, the mitochondrial dysfunction is potentiated by Aβ binding to the mitochondrial membranes and altering their function and dynamics [32,33], leading to synaptic dysfunction [34,35].

Increased levels of iron, copper and zinc, found using immunohistochemistry or proton-induced X-ray emission in close proximity to amyloid plaques in brain tissues of AD patients [36,37], further increase oxidative stress. Especially zinc, normally a component of proteins involved in the antioxidant defenses, can modulate autophagy and activate protein kinase C, NADPH oxidases, extracellular signal-regulated kinase (ERK) 1/2 and poly ADP-ribose polymerase (PARP) and thereby lead to oxidative neuronal damage [38,39]. Binding of metal ions to proteins, amyloid beta or tau promotes protein aggregation and misfolding as well as tau hyperphosphorylation [40,41,42].

Finally, activation of microglia in response to the deposition of extracellular amyloid plaques represents another potential source of ROS [43] mainly as a consequence of activation of microglial NADPH oxidase [44], resulting in production of inflammatory cytokines and chemokines [45]. Moreover, in response to Aβ-induced damage, astrocytes are activated and increase the expression of glial fibrillary acidic protein, which is a marker of astrogliosis [46,47].

The main targets of the excess oxidative species are:

- proteins, the cysteine residues being particularly sensitive to oxidative damage [21,48]
- cellular and organelle membranes, with alterations in ceramide and cholesterol metabolism, peroxidation of membrane lipids [49,50,51] which alter the structure and fluidity of the plasmalemma interfering with the organization and function of signaling pathways, dendritic spines, as well as with the localization and trafficking of receptors [52].
- calcium homeostasis, with increases in intracellular calcium and activation of a series of enzymes, transcription factors such as nuclear factor κB (NF-κB), as well as release of excitatory neuromediators.

ROS, Aβ, and tau protein affect independently and synergistically the activity of *N*-methyl-d-aspartate (NMDA) receptors [53] which, in coordination with α-amino-3-hydroxy-5-methyl-4-isoxazolepropionic acid (AMPA) receptors, regulate the excitatory synaptic transmission and plasticity in the brain playing an essential role in learning and memory [16]. In AD, in addition to the age-related decline of the activity of NMDA receptors, the expression of Aβ further reduces the amount of surface NMDA receptors and, by binding of soluble Aβ to AMPA receptors, promotes the internalization of the latter leading to synaptic dysfunction, loss of dendritic spines, and affecting synaptic plasticity [54,55].

Furthermore, the Ca^2+^/calmodulin complex regulates the expression of Ca^2+^/calmodulin-dependent protein kinase II (CAMKII), a fundamental synaptic protein which is essential for the transition of memories from hippocampal-dependent short term to cortical-dependent long-term storage of memories through its autophosphorylation [56]. In AD the subcellular localization of CAMKII is altered, with elevated cytosolic and reduced synaptosomal levels, which may be responsible for the impaired excitatory synaptic transmission [57,58].

In addition, increased intracellular production of ROS can induce autophagy, a process by which cells degrade cytoplasmic proteins and organelles. In familial forms of AD mutated or absent presenilin-1 impairs the processing of the subunits of the lysosomal pump and compromises the acidification of lysosomes, thereby impairing the autophagic/lysosomal/endosomal system, which significantly contributes to the pathogenesis of dementia [59,60].

Not to be neglected are the effects of ROS on the blood brain barrier (BBB) and the neurovascular unit, an entity composed of neurons, astrocytes and endothelial cells, with a crucial role in maintaining the integrity and activity of the brain by adapting the blood flow to the local demands depending on the activity of the different regions [61]. Oxidative damage often disrupts the BBB [62], a “leaky” BBB being believed to underlie the development of a number of neurodegenerative diseases, AD included [63,64], and leads to dysfunction of the neurovascular unit causing a global reduction in cerebral perfusion and resulting in cognitive impairment [65]. Alterations of the permeability of the BBB have been demonstrated in the human hippocampus during normal aging but in patients with mild cognitive impairment the BBB dysfunction is more pronounced [66]. Following the disruption of the neurovascular unit and BBB breakdown several neurotoxic molecules can enter the brain. In addition, there is inadequate nutrient delivery to the cerebral parenchyma and abnormal expression of growth factors and vascular receptors, which all impair normal neuronal and synaptic function [67].

## 5. Oxidative Stress in the Conditions Known as Risk Factors for Dementia

### 5.1. Oxidative Stress in Cerebrovascular Disease

A growing body of evidence supports the idea of cerebrovascular disease contributing to the pathological mechanisms of AD, leading some researchers to suggest that cerebrovascular disease (CVD) puts an individual at even higher risk of AD than aging [68]. In fact, less than 10% of individual develop pure vascular dementia [69]. Published meta-analyses have shown that ischemic stroke approximately doubles the risk of dementia in older adults and that a patient with stroke has a 59% increased risk of developing AD compared with controls [70,71,72] although it is difficult to determine the extent to which cognitive impairment can be attributed to stroke versus concomitant AD.

Aside from the increased oxidative stress caused by the subjacent risk factors which contributed to the occurrence of an ischemic stroke, after an ischemic insult to the brain there is a sudden burst in the production of free radicals. Due to the significant decrease in regional cerebral blood flow, the reduced mitochondrial electron transport chain activity fails in providing adequate amounts of adenosine-5′-triphosphate levels resulting in neuronal membrane depolarization and sodium and calcium entry into cells. The enhanced neuromediator release (especially of glutamate) further increases the cytosolic calcium levels, which, in turn, lead to mitochondrial dysfunction and generation of ROS [73,74] which activate proteases, phospholipases, and endonucleases.

Restoring blood flow, either spontaneously, as occurs in up to 50% of cases in the first 3–4 days after embolic stroke [75], or through recanalization therapies, further increases oxidative stress by providing oxygen used in many enzymatic oxidative reactions in the mitochondria, subcellular organelles and cytosolic compartments [76]. A large number of studies have shown significant increases in free radical production following an ischemic stroke [77,78,79,80]. In addition, recombinant tissue plasminogen activator itself increases oxidative stress, damages the BBB and can promote hemorrhagic complications [81].

Early neurological deterioration after acute ischemic stroke or after recanalization therapies affects up to one third of patients, among the identified risk factors being old age, hyperglycemia, a larger penumbral area identified by a larger PWI/DWI mismatch (a difference between the affected brain areas outlined by perfusion- and diffusion weighted imaging), large infarctions, or the presence of inflammatory markers [82,83,84]. Another possible mechanism might be increased oxidative damage in the penumbral area [18], as suggested by the more favorable outcome of patients treated with edaravone, a powerful antioxidant, in addition to recanalization methods [85]. The sudden burst of the production of ROS after an ischemic stroke may also contribute to the earlier onset of dementia in stroke survivors [86].

### 5.2. Oxidative Stress, Hypertension and Dementia

A history of hypertension in midlife or late life enhances the neuropathology of AD with abundant amyloid plaques and neurofibrillary tangles in the brain and atrophy of the hippocampus [15]. However, the link between hypertension and dementia is more complex. The accumulation of amyloid beta (Aβ) before the onset of clinically symptomatic dementia could induce hypertension [87]. Moreover, angiotensin-converting enzyme (ACE) has been shown to be able to degrade Aβ in vitro [88] although animal models suggest that treating hypertension with an ACE inhibitor does not increase the amyloid burden in vivo [89]. Several studies have demonstrated excessive production of ROS in essential hypertensive patients and animal models of hypertension [90,91] supporting the idea that increased vascular oxidative stress could be involved in the pathogenesis of essential hypertension [92]. ROS are formed from a variety of sources in the blood vessels, such as NADPH oxidase (NOX), up-regulated in hypertension by humoral and mechanical signals and mainly by angiotensin II [93], uncoupling of endothelial nitric oxide synthase (the resulting nitric oxide reacting with the superoxide anion formed by NOX to generate peroxynitrite) [94], elevated levels of xanthine oxidase [95], as well as the activity of the mitochondrial complex I [96].

The generated ROS act as signaling molecules by causing posttranslational modifications such as oxidation and phosphorylation of proteins which function as receptors, ion transporters, transcription factors, or cytoskeletal proteins [97] and stimulate members of the mitogen-activated protein kinase (MAPK) family such as ERK1/2, JNK, and p38MAPK [98], activate transcription factors such as NFκB, signal transducer and activator of transcription (STAT) activator protein-1 (AP-1), or hypoxia-inducible factor 1 (HIF-1), activate proinflammatory genes, increase cytokine and chemokine production, and increase recruitment and activation of immune cells that will lead to cardiovascular and renal inflammation and fibrosis [92,99] ultimately resulting in hypertension-induced target-organ damage. Associated conditions such as atrial fibrillation, cardiac arrhythmias, heart failure, or cardiac arrest reduce cerebral perfusion and lead to neuronal damage and cognitive decline [100,101] in addition to being prominent risk factors for strokes [102].

### 5.3. Oxidative Stress in Diabetes Mellitus Type 2

Diabetes mellitus is estimated to have affected 427 million people worldwide in 2017, of which over 85% had type 2 diabetes [103], a condition which in the end shows insulin insufficiency.

The strong epidemiological link between diabetes and dementia, particularly AD, is known since the 1990s [104] with an estimated 60% higher risk of diabetic patients to develop dementia [105] as compared to age-matched normoglycemic individuals. It is true that diabetes and AD share common risk factors such as age, sedentary lifestyle, or obesity. However, insulin receptors are expressed throughout the central nervous system regulating neuronal plasticity and synaptic density [15] and AD patients have been shown to exhibit decreased sensitivity and level of insulin receptors compared to controls [106]. Downstream insulin signaling pathways which are altered in AD involve increased expression of mitogen-activated protein kinase (MAPK), disturbed signaling of protein kinase B (Akt), and activation of glycogen synthase kinase 3β (GSK-3β) which hyperphosphorylates tau [107]. Deficiencies in the activity of glucose transporters GLUT-1 and GLUT-3 (which transport glucose across the blood brain barrier) have also been found in AD [108], correlating with decreased glucose metabolism especially in areas affected by AD pathology [109].

The persistent hyperglycemic state in diabetes is associated with overproduction of ROS leading to the vascular and multiorgan complications [110]. Mitochondria have a crucial role in diabetes as well because the increased input of metabolic substrate overwhelms the electron transport system, resulting in ROS overproduction [111]. Hyperglycemic patients or animals frequently exhibit mitochondrial swelling or accumulation of small mitochondria [112], the changed morphology contributing to the increased ROS production (especially superoxide) in hyperglycemic conditions [113]. These ROS have been implicated in the five major pathways leading to vascular complications [114]:

- increased flux of glucose and other sugars through the polyol pathway,
- increased intracellular formation of advanced glycation end-products (AGEs),
- increased expression of the receptors for advanced glycation end products and their activating ligands,
- activation of protein kinase C (PKC) isoforms, and
- overactivity of the hexosamine pathway.

Increased flux through the polyol pathway depletes cells of nicotinic acid adenine dinucleotide phosphate (NADPH), which is an important cofactor required to regenerate reduced glutathione (GSH) and scavenge the excess ROS [114]. Advanced glycation end products (AGEs) resulting from the non-enzymatic reaction of glucose with proteins, and the binding of modified proteins to the AGE receptors (RAGEs) further increases oxidative stress and thereby activates NFκB, modifying gene expression [115]. Hyperglycemia also activates several isoforms of protein kinase C (PKC) and MAPK, further downstream events leading to pericyte apoptosis and inhibition of the expression of endothelial nitric oxide synthase [116,117].

### 5.4. Diet, Obesity, Oxidative Stress and the Risk of Dementia

It has been known for several years that a diet consisting of moderate amounts of saturated fatty acids from meat and poultry, moderated amounts of dairy products such as cheese and yoghurt, moderate amounts of alcohol, especially wine, and rich in vegetables, fruits, cereals, fish, and unsaturated fatty acids, known as the Mediterranean diet, reduces the risk of developing cognitive impairment and AD [118,119]. A 3-year study evaluating the effects of a high adherence compared to a low one to the Mediterranean diet on the AD imaging biomarkers in cognitively normal participants aged between 30 and 60 years concluded that a high adherence confers an average of 1.5–3 years protection against AD [120].

The effect has been explained by reduction of oxidative stress conferred by several compounds of olive oil, like polyphenols [121] or oleuroperin aglycone, which decreases the amount of aggregated proteins, decreases inflammation, and induces autophagy [122,123]. However, recent studies have questioned the association between diet and the risk for dementia [124].

Each meal (glucose intake) of healthy subjects is followed by a temporary increase in ROS generation by polymorphonuclear and mononuclear leucocytes [125]. Ingestion of fat also leads to an extended increase in ROS generation, while ingestion of proteins is associated with a lesser extent of oxidative imbalance [126]. The intensity of postprandial oxidative stress is significantly affected by the amount of caloric intake, since high-caloric meals result in high levels of glucose, triglycerides and free fatty acids entering the blood, which overwhelm the oxidative phosphorylation capacity of mitochondria leading to increased transfer of single electrons to molecular oxygen and increase of superoxide anions entering the circulation [127,128]. Associating antioxidant nutrients, such as orange juice or red wine, decreases the magnitude of postprandial oxidative stress [129,130]. Cooking methods may also influence postprandial oxidative stress: high protein and fat-containing foods cooked at high temperatures form dietary AGEs which can cause acute postprandial endothelial dysfunction [2,131].

Caloric restriction has been shown in animal models as well as in human subjects to slow down the metabolism and associate a reduced oxidative challenge by triggering active defense programs to reduce oxidative stress, a mechanism by which caloric restriction could prolong lifespan [132,133,134]. These include activation of the mitochondrial sirtuin 3 (SIRT3) and nuclear SIRT1 [135,136], of AMP-activated proteinkinase (AMPK) which inhibits acetyl-CoA carboxylases (ACC1 and ACC2), thereby decreasing NADPH consumption in fatty-acid synthesis [137,138] and inhibition of the mammalian target of rapamycin (mTOR) pathway, thereby activating autophagy and recycling of damaged mitochondria [139].

Observational studies have shown that the daily caloric intake has steadily risen in the past 50 years worldwide by about 500 calories/day, although unequally across the world, with a tendency to a plateau at about 3000–3500 calories/day in Europe and Northern America and a steady increase in Asia, Africa, and South America [140]. Unfortunately, this increase in calorie intake is mainly ascribable to higher consumption of edible oils and sweetened beverages due to increased reliance upon processed foods and increased away from home intake [141,142], a trend often referred to as a shift toward the “Western diet’, which has led to a global epidemic of overweight and obesity, hypertension, diabetes, as well as neurodegenerative diseases.

### 5.5. Oxidative Stress and Physical Activity

Clinical studies have shown that aerobic physical activity can improve cognitive function in aging individuals as well as in patients with mild cognitive impairment or AD although no reports on amyloid deposition or other biomarkers of AD were provided [143,144]. Some explanations of these effects have emerged from studies performed on animal models of AD. Exercise has been found to decrease the activity of secretase beta-site APP cleaving enzyme 1 (BACE1) and amyloid precursor protein (APP) compared to sedentary rats [145], decrease caspase-3 expression [15], and up-regulate c-Fos, an indicator of neuronal activity [146]. Another mechanism by which exercise may improve cognition could be an increase in brain derived neurotrophic factor (BDNF) which enhances neurogenesis and dendritic expansion [147] and activates α-secretase, thereby reducing the amount of Aβ peptides [148].

As for oxidative stress, physical activity lowers lipid peroxidation in the elderly to levels found in young sedentary individuals, demonstrating the role of regular exercise in decelerating the aging-associated impairments [149,150]. The low and/or moderate amounts of ROS produced during regular muscle activity act as signaling molecules to upregulate the endogenous antioxidant defense mechanisms in various tissues [151]. Increases in expression of either Cu/Zn superoxide dismutase (Cu/Zn-SOD) or Mn-SOD have been shown, depending on the intensity of exercise [152]. In addition, exercise increases glutathione peroxidase activity in various tissues and, through changing shear stress, activates the expression of vascular NADPH oxidase [153,154].

## 6. Antioxidants in the Treatment of Alzheimer’s Disease

Since the discovery of free radicals and their involvement in the cellular and tissue damages related to aging [14] and the subsequent research proving the implication of oxidative stress in the pathogenesis of cardiovascular disease, neurodegeneration, diabetes or cancer, an increasing number of individuals, especially in developed countries, use antioxidant supplements in an attempt to prolong health and lifespan. Ingestion of antioxidant supplements by the United States population has increased steadily since the mid-1950′s from one percent or less to 40–50 percent today, which translates in costs of 4–5 billion dollars/year [155]. Unfortunately, this approach did not prove successful since many people take supplements to compensate for unhealthy lifestyles. The most commonly used supplements are vitamin E and C [156], coenzyme Q10 [157], melatonin [158], or alpha-lipoic acid [159].

Cellular oxidation-reduction reactions are normally kept under strict control and low concentrations of ROS and reactive nitrogen species act as important signaling molecules in various pathways. For example, the MAPK pathways regulated by ROS induce the production of factors that act on DNA repair, stop the proliferation of injured cells, activate the immune system, and participate in inducing inflammatory responses [160]. Nitric oxide acts in biological signaling in processes such as blood pressure regulation, smooth muscle activity and neurotransmission [161]. In addition, antioxidants are two-edged swords, since trace elements with antioxidant properties such as selenium or copper may become pro-oxidant [162] as may happen with vitamins A, C, and E as well [163].

Many trials with antioxidant supplementations have been undertaken in search for effective means to prevent or delay the onset of AD and other types of dementia, but meta-analyses have shown that the evidence for efficiency so far is only limited [164,165] and most authorities do not recommend antioxidant supplementation but insist on a healthy lifestyle and a balanced diet [166].

Several vegetables rich in natural antioxidants have been shown to act not only through a single mechanism, but to trigger numerous pathways by which they can prevent neurodegeneration [167]. This may be a promising approach in the future, since it became clear that the “one disease-one target–on drug” paradigm has shown serious limitations for the multi-factorial diseases that are dominating our era [1]. Moreover, an antioxidant diet can modulate brain plasticity and build an “epigenetic memory” able to promote neuronal resilience against stressors [168].

Due to the multitude of nutraceuticals (a term including the whole of non-pharmaceutical compounds that may have an impact on health and disease states, general well-being and performance) [169] with demonstrated antioxidant properties and their multiple mechanisms of action, it is difficult to provide a comprehensive classification of these compounds. Hence, a brief review of the evidence on antioxidant mechanisms of some of these compounds, divided into phenolic and non-phenolic ones, will be provided in the following section of this paper.

### 6.1. Phenolic Compounds

Polyphenols have a wide range of structures, but the basic monomer in polyphenols is the phenolic ring. They can be classified depending on the strength of the phenolic ring into phenolic acids, flavonoids, stiblins, phenolic alcohols, and lignans [170]. Several phenolic compounds have been shown to protect against oxidative stress in models of AD and other neurodegenerative disorders.

#### 6.1.1. Resveratrol

Resveratrol is a nonflavonoid polyphenol found in berries, peanuts, and especially in grape seeds and skin [171], and is thought to explain why the moderate consumption of red wine is epidemiologically associated with a low incidence of cardiovascular disease, an association known as the “French paradox” [172].

Aside from enhancing the PI3K/Akt pathway and the nuclear translocation of Nrf2 [173], it has been shown to suppress the activation of NF-κB and MAPK pathways, thereby exhibiting anti-inflammatory effects by attenuating the release of TNF-α and interleukins such as IL-1β in the microglia [174]. In vitro, resveratrol showed protection against beta-amyloid induced toxicity [175] and destabilized Aβ fibrils [176], while in vivo it attenuated neurodegeneration and cognitive impairment [177,178].

Unfortunately, resveratrol is poorly soluble in water, and has a short half-life because upon absorption it is rapidly metabolized in the liver into metabolites with less biological activity [179]. In addition, penetration through the BBB is poor. Therefore, efforts are made to improve its pharmacodynamics and pharmacokinetics by nanoencapsulation in lipid carriers or liposomes, nanocrystals, nanoemulsions, solid dispersions or insertion into polymeric particles. Other researchers have tried to create synthetic derivatives by adding various substitutes such as methoxylic groups, hydroxylic groups or halogens to the aromatic rings of resveratrol [180,181], or to produce synthetic analogues with more potent biological activity [182].

#### 6.1.2. Rosmarinic Acid

Structurally, rosmarinic acid is an esther of caffeic acid and 3,4-di-hydroxy-phenylacetic acid. It is a phenol found in herbs of the mint family, such as rosemary, sage, basil, mint, and thyme [183].

Rosmarinic acid has free radical scavenging activity, boosts GSH synthesis, inactivates NF-κB, increases Nrf2 nuclear translocation, and suppresses lipid peroxidation [184]. In clinical setting, it improved several cognitive performance tasks in healthy young [185] and healthy aged individuals [186]. When tested in patients with moderate AD, Salvia officinalis extract given over 16 weeks showed a significantly better outcome on cognitive functions as compared to placebo [187].

Again, intestinal absorption is less than 1% of the ingested quantity and it crosses poorly the BBB [188], which is why delivery across the BBB must be enhanced with nanoparticles [189].

#### 6.1.3. Curcuminoids

The 3 components of the curcuminoids are polyphenol constituents of turmeric (*Curcuma longa*), belonging to the family Zingiberaceae and used often in Asian cuisine for flavor and color [190]. Epidemiologically, dietary curcumin intake is positively related to cognitive function in healthy elderly individuals [191].

Curcumin has been shown to scavenge free radicals (ROS or reactive nitrogen species) [192], modulate the activity of glutathione peroxidase, catalase and superoxide dismutases [193], and to inhibit ROS-generating enzymes like cyclooxygenases and xanthine oxidase [194]. Curcumin-containing natural dietary supplements were able to diminish lipid peroxidation, the expression of NF-κB, p-ERK, inducible nitric oxide, and cytokines such as IL-1β and IL-6 [195] in mice fed with a high fat diet. In addition, curcumin is able to prevent Aβ aggregation and oligomer formation [196] and, by suppressing β amyloid-induced BACE-1 (beta-site APP-cleaving enzyme) upregulation, diminishes the production of Aβ [197].

However, its lack of solubility in water, high metabolization rate, as well as its poor bioavailability are major limiting factors in the therapeutic application of curcumin [198]. New formulations, such as encapsulation in nanoparticles, liposomes, self-micro-emulsifying delivery systems, or apomorphous solid dispersions are trying to overcome the barriers imposed by the pharmacokinetics of curcumin [199].

#### 6.1.4. Silymarin

Silymarin is a mixture of flavonolignans, flavonoids, and other polyphenolic compounds extracted from milk thistle (*Silybum marianum*), a Mediterranean plant belonging to the Asteraceae family [190]. It has been widely used as a hepatoprotective agent in patients with alcoholic or non-alcoholic fatty liver disease or cirrhosis [200], but in vitro studies have shown that through silybin, a compound of silymarin, it has free radical scavenging properties [201], increases intracellular GSH [202] and SOD levels [203] and protects neuroblastoma cells from Aβ-induced toxicity [204], while in vivo it attenuates memory impairment in mice injected intracerebroventricularly with Aβ [205] and prevents neurodegeneration in mice fed a high fat diet [195]. In addition to the antioxidant activity, it exhibits also anti-inflammatory activity, inhibits amyloid-β aggregation and neuronal apoptosis [206].

Although with a good safety profile, its therapeutic use may be limited by the poor oral bioavailability with poor absorption and rapid metabolism [207].

#### 6.1.5. Chlorogenic Acid

Chlorogenic acid is the main phenolic component in coffee but is also found in beverages prepared from herbs, fruits, or vegetables [190]. In vitro it has been shown to block intracellular ROS accumulation, GSH depletion, and activation of α-secretases, BACE-1, or MAPK [208], while in animal models of AD coffee polyphenols prevented or reduced memory impairments and cognitive dysfunction by reducing amyloid plaques in the hippocampus [209]. In humans, epidemiological studies have linked increased coffee intake to lower risk of dementia and AD, lower Aβ positivity [210,211], while in clinical trials chlorogenic acid supplementation for 16 weeks improved motor and psychomotor speed, executive function and scores on shifting attention tests in clinically healthy participants but who showed increased levels of apolipoprotein A1 and transthyretin, both of which are biomarkers for early-stage cognitive decline [212].

#### 6.1.6. Quercetin

Quercetin, belonging to the flavonol class of flavonoids, is one of the most potent plant origin antioxidants found in watercress, cilantro, lettuce, asparagus, onions, pepperoni, and a variety of berries [213]. The dietary intake of quercetin is around 10–16 mg/day, while the recommended dose of quercetin aglycone as a dietary supplement is 1 g/day [214].

As an antioxidant, quercetin acts as a free radical scavenger [214], induces the Nrf2-ARE pathway [215] and thereby upregulates glutathione transferase, glutathione peroxidase, SOD, catalase and thioredoxin [214]. In addition, it inhibits BACE-1, decreases the phosphorylation of tau protein and the formation of neurofibrillary tangles in transgenic mouse models of AD [216], stimulates mitochondrial biogenesis [217] and inhibits neuroinflammation by inhibiting the production of inflammatory cytokines, reducing nitric oxide production and inducible nitric oxide synthase gene expression in the microglia [214,218].

Quercetin is poorly soluble in water unless a glycosyl group is added [217], is poorly absorbed, rapidly metabolized, and hardly crosses the blood brain barrier, so its therapeutic use is conditioned by the development of related molecules with improved bioavailability [219].

#### 6.1.7. Other Polyphenols

The list of natural phenolic compounds to exhibit anti-inflammatory and antioxidant properties is very long, including:

Sulfuretin, a flavonoid glycoside in the stem bark of *Albizia julibrissin* and the heartwood of *Rhus verniciflua*, suppresses ROS production and enhances the PI3K/Akt pathway and the nuclear translocation of Nrf2 [173].

Anthocyanins, found in Korean black beans, as well as in *Arbutus unedo* (strawberry tree) and lowbush blue-berries (*Vaccinium augustifolium*) [220] also activate the PI3K/Akt/Nrf2 pathway and increases the levels of SOD, GSH and protect in vivo cells from Aβ-induced toxicity [221].

Tea polyphenols attenuated oxidative stress and mitochondrial dysfunction by acting on the same pathway and increasing the levels of SOD, glutathione peroxidase, catalase. In addition, they activate also the tirosin kinase B/CREB/BDNF pathway [222].

Rutin, a flavonoid isolated from buckwheat, was shown to increase phosphorylation of PI3K, Akt, and GSK-3β as well as the nuclear translocation of Nrf2, thereby suppressing GSK activity and diminishing lipid peroxidation. It also modulated neuroinflammation by decreasing the expression of IL-1b and IL-6 [223].

8-hydroxydaidzein, an isoflavone from fermented soy, was found to activate the Nrf2 antioxidant and the Akt/NF-κB anti-inflammatory pathways, to quench ROS and inhibit the production of IL-6 and TNF-α in microglial cells [224].

### 6.2. Non-Phenolic Compounds

A series of natural non-phenolic compounds have also been shown to have neuroprotective effects in AD and other neurodegenerative disorders through their antioxidant and anti-inflammatory properties:

A compound isolated from the marine-derived fungus *Aspergillus* sp. SF6354, known as TMC-256C1, protected microglial cells and mouse hippocampal cells from glutamate-induced neurotoxicity by upregulating the PI3K/Akt/Nrf2 and heme-oxyge-nase-1 (HO-1) pathways, and suppressed the expression of pro-inflammatory markers such as NF-κB or inflammatory cytokines like TNF-α, IL-1β, IL-6, or IL-2 [225].

Acerogenin A, a compound isolated from the stem bark of *Acer nikoense*, suppressed the production of ROS and upregulated the PI3K/Akt/Nrf2/HO-1 pathway thereby protecting cells from glutamate-induced oxidative injury [226].

3,3′-diindolylmethane, a metabolite of indole-3-carbinol present in plants from the Brassicaceae family, was found to attenuate oxidative stress by activating the NF-κB/Akt pathway and have anti-apoptotic effects by attenuating the expression of cytochrome c, caspase-3, and of apoptosis inducing factor AIF [227].

Sulforaphane, isolated from broccoli, cauliflower, or Brussel sprout, modulates the nuclear erythroid 2-related factor 2/antioxidant response element (Nrf2/ARE) pathway, inhibits NF-κB and upregulates neurotrophin expression in animal models of Alzheimer’s disease [228].

Polysaccharide extracts of *Perilla frutescens* enhanced the expression of heme oxygenase-1 and SOD, suppressed the expression of cytochrome c, caspase-3, -8, and -9 and enhanced the expression of Bcl-2 and poly (ADP-ribose) polymerase [229].

Table 1 summarizes the main antioxidant compounds and their mechanism(s) of action in AD.

## 7. Clinical Trials with Natural Antioxidants for Prevention or Treatment of Cognitive Decline or Alzheimer’s Disease

Some of these compounds have been or are evaluated in clinical trials, as shown by a search on the internet in the ClinicalTrials.gov database, setting “all studies” as status, “Alzheimer’s disease” as condition and each of the described dietary antioxidants as other terms followed by the accessing of each study shown as search result [230]:

### 7.1. Curcumin

The effect of 24-week curcumin supplementation has been evaluated in a phase 2 randomized, double-blind, placebo-controlled trial (NCT 00099710) with inconclusive results [231]. Another pilot study evaluated the effect of curcumin and Ginkgo extracts over 6 months on progression of AD (NCT00164749), but no results were published. The status of a phase 2 trial planned to enroll patients in India, looking at the effect of curcumin formulations in AD (NCT01001637) is listed as unknown, while a currently ongoing but no longer recruiting trial, NCT01811381, will evaluate the effect of curcumin and yoga in patients with mild cognitive impairment.

### 7.2. Resveratrol

A phase 2, double-blind, placebo-controlled study (NCT01504854) has evaluated the effect of resveratrol supplementation in patients with probable AD but no published results in peer-reviewed journals are available. Another completed study, NCT00678431, evaluated the effect of dietary supplementation with resveratrol, glucose and malate on the slowing of the progression of AD, but the differences between the active and placebo arms did not reach statistical significance [232]. However, trial NCT02502253 is a phase 1 study currently recruiting patients to demonstrate bioavailability and BBB penetration of a grape seed extract assessed as potential therapy in mild cognitive impairment or prediabetes and type 2 diabetes mellitus.

### 7.3. Quercetin

The SToMP-AD trial (Senolytic Therapy to Modulate Progression of Alzheimer’s Disease, NCT04063124) is currently recruiting patients in a phase I/II trial to assess bioavailability and safety of dasatinib + quercetin in the treatment of older adults with early stage AD [230].

### 7.4. Sulforaphane

The efficacy and safety of sulforaphane in patients with mild to moderate Alzheimer’s disease are evaluated in a currently recruiting randomized, double-blind, placebo-controlled trial (NCT04213391) estimated to be completed by the end of 2022 [230].

### 7.5. Soy Isoflavone

The effects of soy isoflavone has been evaluated in trial NCT00205179, a randomized, placebo-controlled, double-blind pilot study which enrolled 72 participants, and whose findings support the modest cognitive benefit of isoflavones in patients with AD [233].

### 7.6. Olive Oil

Olive oil will be evaluated in 3 studies: The Auburn University Research on Olive Oil for Alzheimer’s Disease (AU-ROOAD), or NCT03824197, enrolled 30 participants and will evaluate the effect of extra-virgin olive oil on brain function (assessed by fMRI imaging), and BBB function (assessed by dynamic contrast-enhanced MRI) as well as on cognitive function and selected biomarkers. Another randomized, double-blind pilot study on 20 healthy participants (NCT04559828) will assess the attenuation of inflammatory processes associated with Alzheimer’s disease after consumption of Pomace olive oil. Finally, another pilot study (NCT04229186) will assess the effect of 12-month olive oil consumption on cognitive performance and biomarkers of neurodegeneration in blood and CSF in 24 patients with mild cognitive impairment and Alzheimer’s disease (EVOCAD) [230].

### 7.7. Caffeine

The University of Lille planned the CAFCA study (NCT04570085), a multicenter, randomized, double-blind, placebo-controlled trial evaluating the effect of a 30-week caffeine treatment on cognition in early and moderate stages of Alzheimer’s disease [230].

## 8. Concluding Remarks

The above list is only a short characterization of the most common dietary antioxidants with beneficial effects in AD. The frustration caused by the many failures of trials with synthetic drugs targeting a specific pathway in AD (and generally in neurodegenerative diseases) boosted the search for new, multitargeted therapeutic approaches. Currently we have only 4 approved drugs for AD which relieve symptoms only temporarily and slow down disease progression but neither of which is disease-modifying [234].

However, natural products have failed as well in showing impressive results so far. Possible reasons for this failure could be:

1. Poor bioavailability, most of the aforementioned molecules being poorly absorbed and rapidly metabolized
2. Despite solid evidence that oxidative stress plays a significant role in neurodegeneration, the targets for pharmacological treatment remain to be identified [21], especially since ROS are involved in complex signaling pathways
3. Randomized placebo-controlled trials are difficult to carry out because a true placebo, meaning a nutrient-deficient group, is unethical to consider [171]
4. It could be that research has focused mainly on the effects of different molecules on neuronal cell lines. Astrocytes outnumber neurons in the human brain and have a key role in defense against oxidative and nitrosative stress, energy storage, mitochondria biogenesis, and synapse modulation [235] as recently shown for amyotrophic lateral sclerosis, where knockout of astrocyte activating factors slows the progression of the disease in a mouse model of ALS [236]. Interfering with the pathological processes in astrocytes could improve neuronal survival in other neurodegenerative diseases as well.
5. Another possible reason is the time point at which treatment is instituted. When clinical symptoms of cognitive impairment are present, the brain has already undergone significant changes in metabolism and Alzheimer pathology has developed, meaning that it may be too late for these dietary compounds to stop or even reverse the upregulated noxious pathways which lead to neurodegeneration and apoptosis.

Until the bioavailability of these natural antioxidant compounds will be improved and important questions as to when to start treatment and what doses to use will be answered, it is of paramount importance to promote a healthy lifestyle and diet, especially among children and young individuals, to take advantage of the globalization and diversify our diet introducing foods with antioxidant compounds from all over the world, an approach that will probably improve our overall health and delay or possibly even avoid the onset of dementia.

## Figures and Tables

**Table 1:** Natural antioxidants with potential benefits in the prevention and/or treatment of Alzheimer’s disease. ↑-upregulation; ↓-inhibition; PI3K-Phosphoinositide 3-kinases; Akt-protein kinase B; Nrf2-nuclear factor erythroid 2–related factor 2; ARE-antioxidant responsive element; NF-κB-nuclear factor kappa-light-chain-enhancer of activated B cells; MAPK-mitogen-activated protein kinase; GSH-glutathione; GPX-glutathione peroxidase; SOD-superoxide dismutase; COX-cyclooxygenase; XO-xanthine oxidase; iNOS-inducible nitric oxide synthase; BACE-1-beta-site APP-cleaving enzyme; CREB-cAMP-response element binding protein; BDNF-brain-derived neurotrophic factor; AIF-apoptosis-inducing factor.

Class	Antioxidant Compound	Main Sources	Antoxidant Mechanism in AD
Polyphenols	Resveratrol	Berries, peanuts, grapes (seeds and skin)	↑ PI3K/Akt pathway ↑ Nrf2 nuclear translocation ↓ NF-κB and MAPK pathways
	Rosmarinic acid	Rosemary, mint, sage, thyme, basil	↑ GSH synthesis, ↓ NF-κB ↑ Nrf2 nuclear translocation
	Curcuminoids	Turmeric	Free radical scavenger ↑ GPX, catalase and SOD ↓ COX and XO, ↓ NF-κB. iNOS, ↓ BACE-1
	Silymarin	Milk thistle (*Silybum marianum*)	Free radical scavenger ↑ GSH, SOD
	Chlorogenic acid	Coffee, herbal/fruit beverages	↑ GSH, ↓ MAPK pathway ↓ BACE-1
	Quercetin	Onions, tomatoes, fruits, green leafy vegetables	Free radical scavenger ↑ Nrf2/ARE, ↓ BACE-1
	Anthocyanins	Korean black beans, red onion, red cabbage	↑ PI3K/Akt/Nrf2 ↑ SOD, GSH
	Tea polyphenols	Green tea	↑ SOD, GPX ↑ tirosin kinase B/CREB/BDNF
	Sulfuretin	Chinese medicinal plants (*Rhus verniciflua, Albizia julibrissin*)	↑ PI3K/Akt ↑ Nrf2/ARE
	Rutin	Buckwheat	↑ PI3K/Akt, ↑Nrf2/ARE
	8-hydroxydaidzein	Fermented soy	↑ Nrf2/ARE ↑ PI3K/Akt
Non-phenolic compounds	Acerogenin A	*Acer nikoense*	↑ PI3K/Akt/Nrf2
	3,3′-diindolylmethane	plants from the Brassicaceae family	↓ NF-κB ↓ AIF
	Sulforaphane	broccoli, cauliflower, or Brussel sprout	↑ Nrf2/ARE, ↓ NF-κB ↑ neurotrophins
	TMC-256C1	Isolated from marine fungus Aspergillus	↑ PI3K/Akt/Nrf2 ↓ NF-κB

## Data Availability

Not applicable.
